# MRI to assess response after neoadjuvant chemotherapy in breast cancer subtypes: a systematic review and meta-analysis

**DOI:** 10.1038/s41523-022-00475-1

**Published:** 2022-09-19

**Authors:** L. M. Janssen, B. M. den Dekker, K. G. A. Gilhuijs, P. J. van Diest, E. van der Wall, S. G. Elias

**Affiliations:** 1 Image Sciences Institute, University Medical Center Utrecht, Utrecht University, Utrecht, The Netherlands; 2 Department of Radiology, University Medical Center Utrecht, Utrecht University, Utrecht, The Netherlands; 3 Department of Pathology, University Medical Centre Utrecht, Utrecht University, Utrecht, The Netherlands; 4 Department of Medical Oncology, University Medical Center Utrecht, Utrecht University, Utrecht, The Netherlands; 5 Julius Center for Health Sciences and Primary Care, University Medical Center Utrecht, Utrecht University, Utrecht, The Netherlands

**Keywords:** Cancer imaging, Predictive markers

## Abstract

This meta-analysis aimed to estimate and compare sensitivity, specificity, positive- (PPV) and negative predictive value (NPV) of magnetic resonance imaging (MRI) for predicting pathological complete remission (pCR) after neoadjuvant chemotherapy (NAC) in patients with early-stage breast cancer. We stratified for molecular subtype by immunohistochemistry (IHC) and explored the impact of other factors. Two researchers systematically searched PUBMED and EMBASE to select relevant studies and extract data. For meta-analysis of sensitivity and specificity, we used bivariate random-effects models. Twenty-six included studies contained 4497 patients. There was a significant impact of IHC subtype on post-NAC MRI accuracy (*p* = 0.0082) for pCR. The pooled sensitivity was 0.67 [95% CI 0.58–0.74] for the HR−/HER2−, 0.65 [95% CI 0.56–0.73] for the HR−/HER2+, 0.55 [95% CI 0.45–0.64] for the HR+/HER2− and 0.60 [95% CI 0.50–0.70] for the HR+/HER2+ subtype. The pooled specificity was 0.85 [95% CI 0.81–0.88] for the HR−/HER2−, 0.81 [95% CI 0.74–0.86] for the HR−/HER2+, 0.88[95% CI 0.84–0.91] for the HR+/HER2− and 0.74 [95% CI 0.63–0.83] for the HR+/HER2+ subtype. The PPV was highest in the HR-/HER2- subtype and lowest in the HR+/HER2− subtype. MRI field strength of 3.0 T was associated with a higher sensitivity compared to 1.5 T (*p* = 0.00063). The accuracy of MRI for predicting pCR depends on molecular subtype, which should be taken into account in clinical practice. Higher MRI field strength positively impacts accuracy. When intervention trials based on MRI response evaluation are designed, the impact of IHC subtype and field strength on MR accuracy should be considered.

## Introduction

Neoadjuvant chemotherapy (NAC) is increasingly used to treat early stage breast cancer. In the United States, NAC to treat breast cancer rose from 15.7% in 2005 to 26.0% in 2015^1^. Its use in the Netherlands has increased from 11% in 2005 to 50% in 2017^2^. Since the breast tumor is left in situ during NAC, this approach enables the evaluation of treatment response. After NAC, a proportion of 9.3–67.0% of patients, depending on molecular subtype, have no residual tumor cells in the surgical resection specimen of their breast (pathological complete remission, pCR). This gives treating physicians room to think about de-escalating surgical treatment after NAC for this group. If surgery could be safely omitted after NAC, patients’ pre-operative anxiety and post-operative morbidity could be avoided, as well as health care costs associated with the surgery itself or its side effects. This ‘wait-and-see’ approach may be an interesting alternative strategy.

To be able to make an informed treatment decision after completion of NAC, physicians have to rely on clinical and/or radiological assessment of tumor response. In clinical practice different imaging modalities are used for this purpose. Magnetic resonance imaging (MRI) is the most versatile imaging technique available for the breast and can give information on tumor biology as well as anatomy. Breast MRI is the most accurate imaging modality to assess response during or after NAC^3,4^, with meta-analyses reporting pooled sensitivities ranging from of 0.65 to 0.91 and pooled specificities ranging from 0.81 to 0.88 to predict pCR^4–7^. However, tumors with specific biological properties may present differently on MRI^8–11^. A study by Loo et. al. has shown that tumors with different subtypes based on immunohistochemistry (IHC) also have different patterns of tumor response following treatment and IHC subtype impacts MRI accuracy for assessing response to treatment^12^.

The IHC surrogate of molecular subtype, based on hormone receptor (HR) and human epidermal growth factor receptor 2 (HER2) receptor status, is one of the most clinically relevant primary breast cancer tumor characteristics. The literature on accuracy of MRI after NAC in different IHC subtypes has been systematically reviewed by Yu et al.^13^. However, meta-analyses that have reported on the accuracy of MRI to evaluate response to NAC after NAC often did not take IHC subtype into account^4–6,14–16^, or did not report the MRI accuracy separately for the IHC subtypes^7,17^. As a consequence, the body of evidence for use of MRI to assess response to NAC specifically for the different IHC subtypes is limited due to smaller individual studies, which sometimes report diverging results.

The goal of this systematic review and meta-analysis was to estimate and compare the accuracy of MRI for detecting pCR after NAC in the different IHC subtypes in patients with early-stage breast cancer, using all currently available published evidence. We also explored the impact of other factors on MRI accuracy, especially MRI field strength.

## Results

### Literature Search

**Fig. 1:**
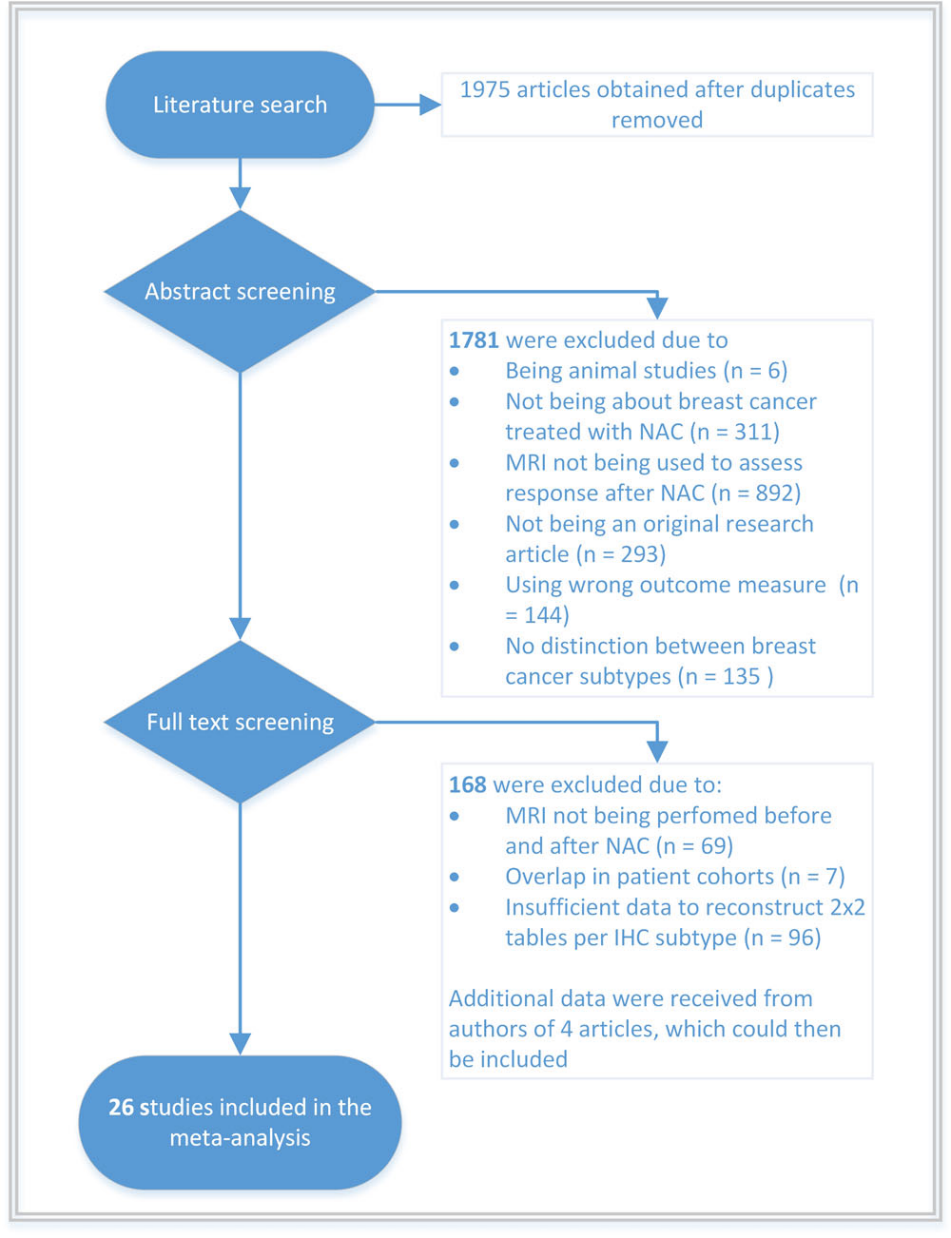
Flowchart of article selection. 1975 abstracts were screened of which 1781 were excluded. 194 full text articles were screened of which 168 were excluded. Four articles could be included again after initial exclusion because the authors provided additional data. 26 studies could eventually be included in the meta-analysis.

A total of 1975 unique articles were retrieved through searches of electronic databases Pubmed and EMBASE after removal of duplicates. Titles and abstracts were screened, after which 1781 articles were excluded for various reasons. For 194 studies, the full text article was carefully reviewed (Fig. 1). Eventually, 26 studies were included in the meta-analysis.

### Included studies

The 26 included studies contained a total of 4497 patients, of whom 2273 had HR+/HER2− tumors, 1156 HR+/HER2+, 1068 HR−/HER2+ and 1658 were diagnosed with HR−/HER2-breast cancer. Patient inclusion in these studies ranged from the years 2000–2019. Different pCR and radiological complete response (rCR) definitions were used across studies. Most of the patients in the studies received a chemotherapy schedule consisting of anthracyclines combined with taxanes. The 26 included studies and their characteristics are listed in Table 1. Additional details on MRI are listed in Supplementary Table 1. The risk of bias of included studies in the different categories of the QUADAS-2 tool was mostly low, although there was some concern for risk of bias in 16/26 studies (Supplementary Fig. 1).

**Table 1:** Summary of included studies.

Author	Year	Design	Inclusion period	Number of HR+/HER2−	Number of HR+/HER2+	Number of HR−/HER2+	Number of HR−/HER2−	Most frequently used chemotherapy regimens included			HER2 directed therapy		MRI field strenght	pCR definition	rCR definition
								Anthra-cyclines	Taxanes	Other agents	Trastuzumab	Pertuzumab			
Eom	2016	Retrospective	2009–2010	.	.	.	64	X	.		.	.	1.5 T	ypT0/is or residual cancer <0.3 cm	NR
Andrade	2017	NR	2005–2012	.	.	40	32	NR	NR	NR	NR	NR	NR	NR	NR
Ramshorst	2017	Retrospective	2000–2016	.	154	143	.	.	X	.	X	X	Mix	ypT0/is	Absence of enhancement
Iwase	2018	Retrospective	2013–2016	53	45	44	36	X	X	.	NR	NR	3.0 T	ypT0	Absence of enhancement
Marin Alcala	2018	NR	NR	.	.	.	114	X	.	.	.	.	NR	ypT0/isN0	NR
Murphy	2018	Prospective	2013–2015	10	.	.	14	X	X	.	.	.	3.0 T	ypT0/is	Disappearance of lesion(s)
Namura	2018	Retrospective	2009–2014	360	95	85	176	X	X	.	X	.	3.0 T	ypT0/is	Absence of enhancement
Gasol Cudos	2019	Retrospective	NR	145	88	64	119	X	X	.	X	X	NR	ypT0/is	Absence of enhancement
Gampenrieder	2019	Retrospective	2006–2016	86	37	33	90	X	X	.	X	X	Mix	ypT0/isN0	Absence of enhancement
Negrao	2019	Retrospective	2014–2017	134	55	31	90	NR	NR	NR	NR	NR	1.5 T	ypT0/is	Absence of enhancement
Zhang, X	2020	Retrospective	2015–2018	11	.	31	27	X	X	.	X	.	1.5 T	ypT0	Absence of enhancement
De Los Santos	2011	Retrospective	2002–2009	33	12	11	25	X	X	.	X	.	1.5 T	ypT0/is	Absence of mass or enhancement
De Los Santos	2013	Retrospective	2002–2011	327	148	101	155	X	X	.	X	.	NR	ypT0	Absence of mass or enhancement
Hayashi	2013	Retrospective	2003–2008	93	54	66	44	NR	NR	NR	NR	NR	1.5 T	ypT0/is	Absence of enhancement
Sabadell	2014	Retrospective	2006–2012	.	.	.	28	NR	NR	NR	.	.	NR	ypT0/is	Disappearance of lesion(s)
Kim	2015	Retrospective	2009–2012	.	.	.	35	X	X	.	.	.	3.0 T	ypT0/is	Absence of enhancement
Fukuda	2016	Retrospective	2005–2007	161	24	32	44	X	X	.	.	.	1.5 T	ypT0/is	Absence of mass or enhancement
Schaefgen	2016	Retrospective	2006–2011	61	.	.	39	X	X	.	.	.	1.5 T	ypT0	Disappearance of lesion(s)
Bufi	2014	Retrospective	2007–2012	143	28	17	37	X	X	.	NR	NR	1.5 T	ypT0	Absence of enhancement
Santamaria	2019	Retrospective	2015–2017	42	10	18	12	X	X	.			1.5 T	ypT0/is	Absence of enhancement
Zhang, K	2020	Retrospective	2013–2018	400	197	222	212	X	X	X	X	.	1.5 T	ypT0/is	Absence of enhancement
Pasquero	2020	Retrospective	2015–2017	.	.	.	13	X	X	.	.	.	Mix	NR	NR
Graeser	2021	Prospective	2012–2015	.	103	50	91	.	.	X	X	X	Mix	ypT0/isN0	Absence of enhancement
Nakashima	2021	Retrospective	2014–2017	117	28	42	89	X	X	.	X	.	3.0 T	ypT0	Absence of enhancement
Palshof	2021	Retrospective	2016–2019	52	47	22	30	X	X	.	X	.	1.5 T	ypT0	Absence of enhancement
Winder	2021	Retrospective	2013–2018	45	31	16	42	X	X	.	X	.	3.0 T	ypT0	Absence of enhancement in breast and axilla

*NR* not reported.

### Accuracy of MRI in IHC subtypes

**Fig. 2:**
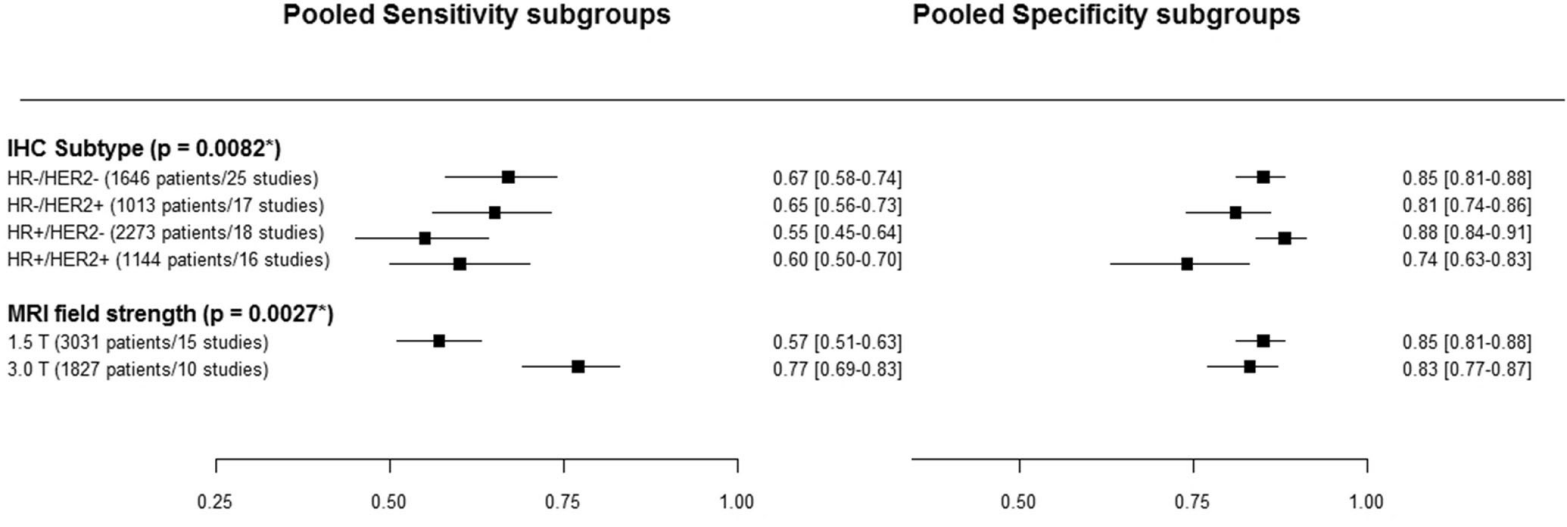
Summary estimates for sensitivity and specificity and 95% CI for subgroups. *P*-values represent results from meta-regression analysis with a model containing only IHC subtype or only MRI field strength.

The pooled sensitivity was highest for the HR−/HER2− subtype at 0.67 [95% conficence interval (CI) 0.58–0.74], 0.65 [95% CI 0.56–0.73] for the HR−/HER2+, 0.60 [95% CI 0.50–0.70] for the HR+/HER2+ and lowest at 0.55 [95% CI 0.45–0.64] for the HR+/HER2− subtype. The pooled specificity was highest for the for the HR+/HER2− subtype at 0.88 [95% CI 0.84–0.91], 0.85 [95% CI 0.81–0.88] for the HR−/HER2−, 0.81 [95% CI 0.74–0.86] for the HR−/HER2+, and lowest at 0.74 [95% CI 0.63–0.83] for the HR+/HER2+ subtype (Fig. 2).

Metaregression analysis showed a significant impact of IHC subtype on MRI accuracy (*p* = 0.0082). Specificity of MRI for detecting pCR was significantly lower in the HR+/HER2+ compared to the HR+/HER2− and HR−/HER2− subtypes (*p* ≤ 0.01). HR−/HER2+ has a significantly lower specificity compared to HR+/HER2− subtype (*p* = 0.046). There was no significant difference in sensitivity between the different IHC subtypes (Fig. 2).

### Exploration of heterogeneity

Visual inspection of the forestplots revealed marked between-study heterogeneity (Supplementary Fig. 2). Deek’s test showed no significant funnel plot asymmetry for any of the subtypes, indicating no signs of publication bias (Supplementary Fig. 3).

Metaregression analysis was additionally conducted for MRI field strength (1.5 T vs. 3.0 T). MRI field strength of 3.0 T was associated with a higher sensitivity compared to 1.5 T (*p* = 0.00063). There was no significant impact on specificity (*p* = 0.41). Figure 2 shows the sensitivity and specificity for MRI with 1.5 T and 3.0 T. A meta-regression model including IHC subtype and MRI field strength had a significantly better fit compared to a model with IHC subtype alone (*p* = 0.002) or MRI field strength alone (*p* = 0.0085). There was no significant interaction between IHC subtype and field strength (*p* = 0.99). The estimates for sensitivity and specificity and the associated 95% CI for each combination of subtype and field strength can be found in Table 2. The positive predictive value (PPV; probability of pCR when rCR is seen on MRI) was lowest in the HR+/HER2− subtype and highest in the HR−/HER2− subtype (Table 2). The negative predictive value (NPV; probability of residual disease on pathology when residual disease was seen on MRI) was highest in the HR+HER2− and lowest in the HR−/HER2+ subtype. Both positive and negative predictive value were higher in 3 T MRI compared to 1.5 T MRI.

**Table 2:** Estimates for each of the scenarios from the meta-regression model including IHC subtype and MRI field strength.

Subtype	MRI field strength	Sensitivity (95% CI)	Specificity (95% CI)	pCR rate (95% CI)*	PPV (95% CI)	NPV (95% CI)
HR−/HER2−	1.5 T	0.61 (0.51–0.70)	0.88 (0.83–0.92)	0.336 (0.309–0.364)	0.73 (0.64–0.80)	0.82 (0.78–0.86)
HR−/HER2+	1.5 T	0.61 (0.51–0.70)	0.81 (0.72–0.87)	0.390 (0.357–0.423)	0.66 (0.58–0.75)	0.76 (0.71–0.81)
HR+/HER2−	1.5 T	0.51 (0.39–0.62)	0.89 (0.85–0.92)	0.096 (0.085–0.108)	0.33 (0.25–0.42)	0.94 (0.93–0.96)
HR+/HER2+	1.5 T	0.55 (0.43–0.66)	0.78 (0.70–0.85)	0.228 (0.203–0.253)	0.43 (0.34–0.52)	0.85 (0.82–0.89)
HR−/HER2−	3.0 T	0.79 (0.70–0.86)	0.86 (0.80–0.91)	0.336 (0.309–0.364)	0.75 (0.67–0.81)	0.89 (0.85–0.92)
HR−/HER2+	3.0 T	0.79 (0.69–0.86)	0.78 (0.68–0.85)	0.390 (0.357–0.423)	0.69 (0.61–0.77)	0.85 (0.80–0.90)
HR+/HER2−	3.0 T	0.71 (0.58–0.81)	0.87 (0.81–0.92)	0.096 (0.085–0.108)	0.37 (0.28–0.47)	0.97 (0.95–0.98)
HR+/HER2+	3.0 T	0.74 (0.63–0.83)	0.75 (0.66–0.83)	0.228 (0.203–0.253)	0.47 (0.38–0.56)	0.91 (0.87–0.94)

*PPV* positive predictive value, *NVP* negative predictive value. True positive is defined as both rCR and pCR. True negative was defined as residual disease on both MRI and pathology. *pCR rates and 95% CI are calculated based on data from the pooled analysis by Cortazar et al.^28^.

PCR definition, rCR definition, and number or patients in the study did not significantly impact MRI accuracy.

Following a sensitivity analysis with the exclusion of different subsets of the data, the precise estimates of sensitivity and specificity varied slightly, but there was no meaningful impact.

## Discussion

To our best knowledge, this is the first meta-analysis to calculate the pooled sensitivity, specificity, PPV and NPV of MRI for detecting pCR after NAC in the different IHC breast cancer subtypes. In addition, we were able to investigate the influence of other factors on MRI accuracy as well. Our results show that MRI specificity differs significantly between subtypes [Table 2 and Fig. 2]. The pooled sensitivity was highest in the HR−/HER2− and lowest in the HR+/HER2− subtype. The specificity was highest in the HR+/HER2− and lowest in the HR+/HER2+ subtype. The projected PPV of MRI for pCR is highest in the HR-/HER2- subtype and lowest in the HR+/HER2− subtype. NPV was highest in the HR+/HER2− and lowest in the HR−/HER2+ subtype. MRI field strength had a significant impact on MRI sensitivity, with a higher sensitivity, PPV and NPV in 3 T MRI compared to 1.5 T.

Other meta-analyses have reported on the overall sensitivity and specificity of MRI for detecting pCR. Some report slightly higher sensitivities compared to our data^4,6,7^, although studies with different field strengths were included in these meta-analyses as well. This discrepancy may be due to differences in inclusion criteria, as we only included studies in which IHC subtype was taken into account, and these meta-analyses did not apply this criterion. The meta-analysis by Virostko et al.^7^, only included studies that investigated DCE MRI and diffusion-weighted imaging, which may explain the higher pooled sensitivity. Liu et al.^5^ found an overall sensitivity of 0.65 (95 %CI: 0.45–0.80) which has more uncertainty but is in line with our results. Specificities in earlier meta-analyses were in the same range of those that we report here.

The difference in MRI accuracy between IHC subtypes could be explained by biological differences between tumor subtypes. These differences may translate into different presentations on MRI. Compared to HER2+ and triple negative subtypes, HR+ tumors more often present as non-mass enhancement and more often reduce into multiple small foci after NAC^12^. This finding is also pathologically validated by a recent study showing that a circumscribed pattern of the residual tumor after NAC was more frequent in HR− tumors and a scattered pattern of response was much more frequent in the HR+ tumors. Triple negative and HER2+ tumors are more often high grade compared to HR+ tumors, and a scattered pattern was more frequent among grade 1 or 2 than among grade 3 tumors^18^. Further, high grade cancers show higher proliferation and are thereby inherently more sensitive to NAC.

The biological differences and related sensitivity to chemotherapy between subtypes also translate into a wide range of pCR rates. Additionally, the introduction of very effective anti-HER2 targeted therapy in HER2+ tumors and increasingly aggressive treatment in triple negative breast cancer also cause an increase in the pCR rates in these subtypes, widening the gap in pCR rates even more. The large differences in PPV between the subtypes can probably be party explained by this variety in pCR rates. Of note: when predictive values would be calculated based on higher pCR rates associated with more effective treatments, the PPV would turn out higher and NPV would turn out lower compared to what is shown here (e.g., in HR−/HER2+ tumors treated with chemotherapy, trastuzumab and pertuzumab or in HR-/HER2- tumors treated with platinum-based chemotherapy).

There could potentially also be a direct impact of treatment regimen on MRI accuracy. One study reports that taxane-based regimens cause more suppression of both tumor- and background enhancement on MRI compared to regimens without taxanes^19^. Consequently, one may speculate that the responses in tumors treated with taxanes are more frequently overestimated on MRI. The impact of adding HER2-directed therapy to NAC on the way breast cancer presents on MRI is largely unknown. One study suggests a lower accuracy of MRI in HER2-positive tumors treated with trastuzumab or pertuzumab, possibly because the angiogenesis of the tumor is reduced by these treatments which may impact contrast uptake^20^. This mechanism could be an explanation for the lower specificity of MRI that we found in the HER2+ groups. Unfortunately, there was limited reporting, too little between-study variation, and too much within-study heterogeneity on treatment regimen in our data to investigate its effect on MRI accuracy.

In addition to subtype, we also found a significant impact of field strength on MRI sensitivity for pCR. Two other, smaller, meta-analyses did not find a significant effect in their meta-regression analysis^4,6^. A possible explanation for a higher sensitivity in 3 T MRI could be the higher spatial resolution and greater contrast-to-noise ratio^21^. The more detailed anatomy may give the radiologist more confidence to rule out residual disease. A higher temporal resolution in 3 T MRI can also give more detailed kinetic information^21^, perhaps also making a distinction between residual tumor and fibrous tissue easier. Although it is possible that 3 T MRI is in fact more sensitive for detecting pCR compared to 1.5 T MRI, this effect may also be caused by a correlation of MRI field strength with other factors in the MRI protocol or differences in interpretation by radiologists.

Our results can give clinicians additional information when interpreting the breast MRI performed for response evaluation after NAC. Our findings are also important when considering future perspectives in breast cancer research. If pCR in the breast after NAC can be accurately assessed non-invasively, breast surgery could perhaps be replaced by a wait-and-see approach^22^. This would spare patients the morbidity of breast surgery and possible psychological impact of losing (part of) their breast. However, if breast surgery is omitted we will also lose the up to now most accurate method to evaluate the response to treatment, since the resection specimen will no longer be available for pathologic assessment. Before such a wait-and see approach can be implemented, the prevention of morbidity needs to outweigh the risk of a missed residual tumor. At this point, the consequences of a missed residual tumor after NAC and subsequent delayed intervention are not clear. These may depend on the chosen surveillance strategy and post-NAC local and systemic treatment.

If one would want to be sure of a pCR before omitting breast surgery, radiological assessment of MRI alone has insufficient PPV to support this decision in either of the subtypes based on this meta-analysis. Response prediction should first be improved, which could possibly be achieved by using more advanced image analysis, (liquid) biopsies, or their combination.

If MRI after NAC is combined with additional methods for detection of residual disease, alternative treatment strategies can be considered. One can envision that the combination of rCR on MRI and negative (liquid) biopsy would decrease the risk of residual disease so that surgery could be omitted. If one of the methods point to possible residual disease, one could also consider leaving out surgery for localized disease, but giving adjuvant treatment for systemic disease. There may also be a role for radiotherapy after omitting surgery.

Even after maximal improvement of response evaluation, it may not be possible to perfectly predict pCR with non-invasive methods, and even pathological evaluation itself is not 100% accurate because of the inherent limited sampling of resection specimens.

Additionally, missed residual disease may not have the same impact in each subtype, as shown in a recent pooled analysis where patients with minimal residual disease after NAC (RCB-I) seem to have comparable prognosis to patients with pCR in the HR+/HER2− group^23^. One study even found that rCR in this group is prognostically more important than pCR^24^. So perhaps future research in this subtype should not focus on improving the prediction of pCR with imaging but rather on the prediction of a more comprehensive surrogate endpoint like RCB or exploring alternative endpoints and methods for prediction.

Strengths of our study include the systematically performed search, screening, and data extraction by two independent researchers and the large and complete dataset. A limitation of our study is the large between-study heterogeneity in sensitivity and specificity that could not be completely explained by the factors investigated. Also, improvements in MRI technique in recent years may not be well reflected, although the exclusion of studies that finished inclusion before 2010 did not lead to significantly different results. In general, standardization of MRI protocols and rCR definitions would be very helpful in this line of research.

An important conclusion from our work is that the accuracy of MRI for pCR after NAC depends on breast cancer IHC subtype. Different breast cancer subtypes have different biology with different MRI phenotypes, and this should be considered in both response evaluation in the daily clinic and future research. Our results also suggest a higher sensitivity to detect pCR in 3.0 T MRI compared to 1.5 T MRI. When intervention trials based on MRI response evaluation are designed, the impact of IHC subtype and field strength on MR accuracy should be taken into account.

## Methods

The protocol for the meta-analysis can be found on PROSPERO under registration number CRD42020221127. PRISMA guideline for reporting on DTA reviews was followed^25^.

### Literature search

The research question for this systematic review and meta-analysis was formulated following DDO(Domain, Determinant, Outcome). The research question was: what is the diagnostic performance of breast MRI after neoadjuvant chemotherapy for detecting pathological complete response in early stage breast cancer patients for the different IHC subtypes?

PUBMED and EMBASE were systematically searched for relevant articles and abstracts. For relevant articles, the reference list was checked. No restrictions were applied on the search, including publication period or language. The search was re-run prior to the final analysis, the date of the last search was 15–7–2021. In the search string, we used a combination of synonyms for breast cancer, neoadjuvant therapy, MRI, and response (Supplementary Table 2).

### Study selection

The inclusion criteria were as follows: (1) women with histopathologic proven early stage, invasive breast cancer treated with NAC; (2) patients who underwent MRI after NAC to assess response to treatment before surgery; (3) patients have undergone breast surgery after completion of NAC and data on pCR are documented; (4) original research article; (5) sufficient data to reconstruct two-by-two (2 × 2) table per IHC subtype. The data used to reconstruct the 2 × 2 tables were: number of cases with pCR, number of cases with rCR, number of cases with both rCR and pCR and number of cases with both non-rCR and non-pCR. For studies that fulfilled inclusion criteria 1–4, but sufficient data to reconstruct 2 × 2 table per IHC subtype or other information was missing, the authors were contacted. Studies investigating breast cancer in men were excluded because of the difference in presentation on MRI. If one study or cohort was reported by more than one publication, only the most informative publication was included. Articles that only reported on one IHC subtype were included. Study selection was performed by two independent researchers (L.M.J. and B.M.D.D.) using Rayyan^26^. Where disagreement arose, this was discussed until consensus was reached.

### Data extraction

We extracted the following parameters from each of the included articles: Title, authors, journal, year of publication, whether or not the study was prospective, whether or not it was a consecutive/random sample, the inclusion period, the number of patients and tumors included, which patients were excluded for the study, what treatment schedule the patients received, the definition of the different IHC subtypes, the numbers of tumors in each subtype group, years of experience MRI reader, the definition of rCR that was used, MRI technical parameters (field strength, manufacturer, description of sequences and coil), the definition(s) of pCR that was/were used, years of experience of the pathologist assessing pathological response, the interval between the post NAC MRI and surgery, and the variables to fill the 2×2 tables for each IHC subtype.

### Data quality assessment

The methodological quality of the included studies was assessed by using the Quality Assessment of Diagnostic Accuracy Studies 2 (QUADAS-2) tool^27^. The risk of bias and applicability concern was determined for each of these domains: patient selection, index test (MRI), reference standard (pathologic assessment surgical resection specimen), and patient flow.

### Data analysis

We categorized tumors based on HR and HER2-receptor status (positive or negative) into 4 subtypes: HR−/HER2−, HR−/HER2+, HR+/HER2−, and HR+/HER2+. Two researchers independently extracted 2×2 tables from the primary studies for each of the subtypes. True positive was defined as having both pCR and rCR. In line with this definition, reported sensitivity estimates should be interpreted as the percentage of patients with pCR in whom the MRI is indeed assessed as rCR, and specificity as the percentage of patients without pCR in whom the MRI is indeed assessed as non-rCR. We used the bivariate random-effects model to obtain pooled sensitivity and specificity including 95% CI. To investigate the influence of IHC subtype on sensitivity and specificity estimates, we used meta-regression analyses. The likelihood ratio test was used to compare the fit of meta-regression models. The projected PPV and (NPV) were obtained by combining the estimated sensitivity and specificity for each of the subtypes with published pCR rates within breast cancer molecular subtypes^28^ and the pooled sensitivity and specificity from our meta-analyses. 95% CI for PPV/NPV were estimated by Monte Carlo simulation (100,000 fold) using the metaregression-based covariance matrix for sensitivity and specificity in combination with published pCR rates and associated uncertainty^28^. Metaregression was also used to explore the impact of MRI field strength (1.5 T vs. 3.0 T). Deek’s test for funnelplot asymmetry was used to assess publication bias. A sensitivity analysis was conducted for each of the IHC subtypes to see the impact of removing studies from the analysis which were (1) at risk of bias or had concerns regarding applicability (2) were a conference abstract without a published article (3) rCR definition was not given (4) pCR definition was not given or (5) if patient inclusion was finished before 2010 to account for improvements in technical aspects of MRI. All reported *P* values were two-sided, and *P* < 0.05 was considered statistically significant. R software v.1.3.1093^29^ was used for all statistical analyses. We mainly used the function ‘reitsma’ and ‘forest’ from package ‘mada’ v.0.5.10 and the function ‘metabin’ and ‘funnel’ from package ‘meta’ v.4.18–0.

### Reporting summary

Further information on research design is available in the Nature Research Reporting Summary linked to this article.

## Supplementary information

Supplementary Material

Reporting Summary

## Data Availability

The data collection forms, datasets containing extracted data, and datasets used for analysis and R code are available from the corresponding author on reasonable request.
