# Supplementary material for: MRI to assess response after neoadjuvant chemotherapy in breast cancer subtypes: a systematic review and meta-analysis
Source: NPJ Breast Cancer. 2022 Sep 19;8:107. doi: 10.1038/s41523-022-00475-1 (PMC9485124; doi:10.1038/s41523-022-00475-1)

Supplementary Table 1: additional information on MRI scanners used in the included studies. NR = not reported

Author	Journal	Year	MRI Field Strength (Tesla)	Manufacturer	Sequence description	Coil description
Eom	AJR	2016	1.5	Siemens	The standard dynamic protocol used for the evaluation of supraclavicular and axillary lymph nodes included the following sequences and parameters: an axial 2D T2-weighted STIR turbo spin-echo pulse sequence (TR/TE/time interval, 6700/74/150; FOV, 300 × 300 mm2; matrix, 448 × 448; and slice thickness, 5 mm), axial unenhanced and contrastenhanced fat-saturated 3D T1-weighted FLASH volume-interpolated breathhold examination pulse sequences (TR/TE, 5.2/2.4; FOV 340 × 340 mm2; matrix, 384 × 384;	dedicated bilateral phase array coil
Andrade F.E.M.	Cancer Res	2017	NR	NR	NR	NR
Ramshorst, M.S.	Breast Cancer Res Treat	2017	1.5 and 3	1.5T Siemens 3T Phil	First, an unenhanced coronal 3D fast field echo (FFE) (thrive) sense T1-weighted sequence was performed. Subsequently, contrast containing gadolinium (0.1 mmol/kg) was administered intravenously followed by dynamic imaging in five consecutive series at 90-s intervals with voxel size 1.21 9 1.21 9 1.69 mm3 (1.5 T) or 1.1 9 1.1 9 1.2 mm3 (3.0 T).	dedicated bilateral phased array breast coil, philips: dedicated 7-element sense breast coil was
Iwase, M.	Breast Cancer	2018	3.0	GE healthcare Signa	pre-contrast and dynamic contrastenhanced imaging. Dynamic study was performed before and 45, 180, and 315 s after starting intravenous gadolinium injection.	breast array coils
Marin Alcala M.	Ann. Oncol.	2018	NR	NR	NR	NR
Murphy, C.	Intern Med J	2018	3T (except for 3 studies at 1.5)	Siemens	a fat-suppressed sequence, a non-fat-suppressed T2 sequence, the dynamic rapid sequence fat-suppressed T1 sequence and a high-resolution fat-suppressed T1 sequence	16-channel AL-dedicated breast coil
Namura, M.	Clin Breast Cancer	2018	3.0	GE Health Care, Siemens	high-resolution precontrast imaging and dynamic contrast-enhanced imaging. It included a localizing sequence followed by unilateral T2-weighted axial imaging (repetition time [TR]/echo time [TE]: 4748/86, slice thickness ¼ 5 mm, field of view [FoV] ¼ 350 mm, matrix ¼ 512 256). After a scout scan, sagittal, unilateral T1-weighted, precontrast images (TR/TE: 5.2/2.2 slice thickness ¼ 1 mm, slice gap ¼ 0 mm, FoV ¼ 350 mm, matrix ¼ 384 330) were acquired. A dynamic study in the axial plane was performed before and 60, 180, 300, and 420 seconds after starting intravenous injection of 1.5 mL/kg of meglumine gadopentetate at the beginning of the fifth acquisition, followed by 10 mL saline for flushing.	dedicated 8-channel (GE Health Care) and 32-channel (Siemens Healthineers) breast array coils
Gasol Cudos A.	Ann. Oncol.	2019	1.5	NR	NR	NR
Gampenrieder, S.P,	Breast Cancer Res	2019	3.0 or 1.5, 11 scans on <1.5T	Philips	axial T2-weighted fat-suppressed images (echo time (TE)/repetition time (TR)/ inversion recovery (IR) of 60/9065/230 msec; with a slice thickness of 3mm and a field of view of 30–40 cm) and axial diffusion-weighted images (TE IR/TR of 59/8157 msec; b-values up to 600; slice thickness 3 mm). Finally, four axial T1-weighted fat-suppressed dynamic acquisitions (TE/TR, 2.3/4.1msec with a slice thickness of 1mm) were registered over the duration of 5 min after intravenous contrast medium injection. As gadolinium contrast agent we used either 15 mL gadoteric acid (DOTAREM®, Guerbet, Cedex, France) or 7mL gadobutrol (GADOVIST®, Bayer Schering Pharma, Berlin, Germany followed by a saline bolus flash. The remaining 11 breast MR exams were performed in external institutes using scanners of less than 1.5 Tesla.	dedicated phased-array breast coil
Negrao	Radiol Bras	2019	1.5	Signa GE or Achieva Philips	dedicated breast coil	
Zhang, X.	Quant Imaging Med Surg	2020	1.5	GE Healthcare	First, sagittal fat-saturated T2-weighted, fast-spin echo images of the bilateral breasts were recorded using the following parameters: repetition time/echo time, 4,040 ms/81 ms; echo train length, 19; slice thickness, 5 mm; slice gap, 1 mm; field of view, 220 mm; matrix size, 320×224; number of excitations, 2; imaging time, 1 min 41 s. Then, unenhanced and enhanced sequences of axial fat-saturated gradient recalled echo T1-weighted images were acquired with the following parameters: repetition time/echo time/inversion time, 6.1 ms/2.9 ms/13 ms; flip angle, 10°; slice thickness, 3.2 mm; slice gap, 0 mm;	resolute 8-channel high-definition breast coil

					field of view, 360 mm; matrix size, 350×350; number of excitations, 0.8; imaging time, 58 s per phase; 8 phases of enhancement. Gadopentetate dimeglumine was administered at a dose of 15 mL and a rate of 2.5 mL/s, followed by a 20-mL saline flush given with an automatic injector.	
De Los Santos, J.	Clin Breast Cancer	2011	1.5	GE	T1 axial, T2 fat-sat axial, pre- and post-contrast sagittal dynamic sequence with four repeats at 90-second intervals, 2- to 3-mm slice thickness, and delayed post-contrast T1 fat-sat axial, color map analysis, and maximum intensity projection	8-channel In Vivo dedicated breast coil
De Los Santos, J. F.	Cancer	2013	NR	NR	NR	NR
Hayashi , Y.	Oncol Lett	2013	1.5	Philips	Transverse images were obtained by diffusion-weighted (DW) imaging. Coronal images were obtained by contrast-enhanced dynamic imaging. Sagittal images were obtained by contrast-enhanced late-phase imaging. Additionally, depending on the case, sagittal images were obtained by T2-weighted fat-suppressed imaging prior to infusion of contrast material.	SENSE body coil
Sabade ll M.D.	Eur. J. Cancer	2014	NR	NR	NR	NR
Kim, M. J.	Acta Radiol	2015	3.0	Trio Tim Siemens	T1-weighted (T1W) non-fat-suppressed axial sequence (repetition time/echo time, 280/2.6 ms; flip angle, 65; bandwidth, 543 Hz/pixel; matrix size, 512512; voxel size, 0.70.73.0 mm), T1W non-fat-suppressed precontrast and 2D dynamic contrast-enhanced (DCE) axial (repetition time/echo time, 280/2.6 ms; flip angle, 65; bandwidth 540 Hz/pixel; matrix size, 512343; voxel size, 1.00.73.0 mm) with intravenous injection of 0.2 cc/kg gadolinium-diethylenetriaminepenta acetic acid (Gd-DTPA, Magnevist; Berlex Laboratories Inc., Montville, NJ, USA) in order to obtain one precontrast set and six postcontrast sets, the temporal resolution was 73 s for each frame, and finally T2-weighted (T2W) turbo spin echo axial images (repetition time/ echo time, 4360/82 ms; flip angle, 150; bandwidth, 305 Hz/pixel; matrix size, 512512; voxel size, 0.70.73.0 mm). The slice thickness was 3mm and field of view was 32–34cm for all of the MRI sequences.	edicated fourchannel breast array coil.
Fukuda, T.	Springe rplus	2016	1.5	GE	Our imaging protocol included a localizing sequence followed by unilateral fast spin-echo T2-weighted coronal imaging (TR/TE, 4800/85 ms; echo train length 16, and matrix 384 × 224) with fat suppression by chemical shiftselective imaging sequences. Other parameters were as follows: field of view, 260 mm; section thickness, 3 mm and interslice gap, 0 mm. This examination was followed by combined dynamic contrast-enhanced unilateral coronal breast imaging. An enhanced T1-weighted examination 3D gradient echo sequence with fat suppression by spectral inversion recovery was performed before and after contrast material injection. The image parameters were as follows: TR/TE/FA, 3.6 ms/1.0 ms/15°; FOV, 26 × 26 cm; matrix, 320 × 240; section thickness, 3.0 mm; interslice gap, 0 mm and acquisition time, 60 s. A dynamic study in the coronal plane was performed before and 60, 120, 180 and 240 s after starting intravenous injection of 0.2 mmol/kg of gadodiamide hydrate (Omniscan®, Daiichi-Sankyo, Tokyo, Japan) at a rate of 3 mL/s, followed by a 20 mL saline flush at the rate of 3 mL/s.	dedicated four-channel breast array coil.
Schaef gen, B.	Ann Surg Oncol	2016	1.5	Siemens	NR	bilateral breast coil (Seven-Channel Biopsy Breast Array
Bufi	Eur J Radiol	2014	1.5	GE	STIR, DWI, 3D fast spolied graideint echo	dedicated breast coil
Santam aria G.	Europe an Journal of Radiolo gy	2019	1.5	GE + Siemens	T2 FSE, 3D t1 weighted, DWI (EPI)	edicated bilateral fourchannel breast surface coil
Zhang	Cancer manag ement and researc h	2020	1.5	Aurora	precontrast axial T2-weighted fat-suppressed sequence (TR 6,680 ms, TE 29 ms, thickness 3 mm) and axial T1-weighted fat-suppressed sequences (TR 4.8 ms, TE 29 ms, thickness 1.1 mm, FOV 360 mm, matrix 360×360×128) before and after a bolus of a gadolinium-based contrast agent (gadopentetate dimeglumine, 0.1 mmol/kg) was injected at a rate of 2 mL/s. Postcontrast images were obtained at 90, 180, 270, and 360 seconds after the injection	breast coil
Pasque ro	In vivo	2020	0.4 and 1.5	Multiple	Open MRI Hitachi 0.4 Tesla, MRI Philips Ingenia 1.5 Tesla or MRI Philips Achieva D-Stream 1.5 Tesla.	NR

Graeser	Breast cancer researc h : BCR	2021	1.5 and 3.0	NR	The standardized imaging protocol of 1.5-T and 3.0-T systems with a dedicated breast multichannel surface coil consisted of an axial bilateral twodimensional multi-section gradient-echo dynamic series (repetition time 250 ms; echo time 4.6 ms (1.5 T) or 2.3 ms (3 T); flip angle 90°) with a section thickness of 3 mm and full 512 × 512 acquisition matrix. The voxel size of all scans was kept constant along all exams with a maximum of 0.8. To ensure a high spatial resolution, the field of view was adapted to the individual breast size of the patient with a minimum of 280 mm and a maximum of 350 mm keeping the voxel size within the intended range. The dynamic sequence was performed prior to and four times after bolus injection of macrocyclic gadolinium agent, gadobutrol (Gadovist®/Gadavist®, Bayer AG, Leverkusen, Germany; 0.1 mmol/kg body weight), followed by a saline flush. Depending on the site preference, fat suppression or image subtraction was used for visualization of enhancement in the T1 gradient-echo sequence. A standard center of k-space between 60 and 90 s was used in all exams. In addition, an axial T2-weighted fast spin-echo sequence without fat suppression and with identical anatomic parameters as the T1-gradient echo sequence was performed.	dedicated breast multichannel surface coil
Nakash ima	Breast cancer (Tokyo, Japan)	2021	3.0	Philips	T1-weighted high-resolution isotropic volume examination sequences were performed using a 3D gradient-echo technique (TR/TE, 3.4/1.8 ms; flip angle, 10°; FOV, 33 × 33 cm; matrix, 400 × 320; section thickness, 1.8 mm interpolated to 0.9 mm) with active fat suppression. Images in the axial plane were acquired before and 20, 85, and 280 s after the injection of contrast material, either 0.1 mmol/kg gadopentetate dimeglumine (Magnevist; Bayer, Osaka, Japan) or gadobutrol (Gadovist; Bayer, Osaka, Japan). In addition, contrast-enhanced unilateral sagittal images with high spatial resolution were obtained for the breast with cancer and the contralateral breast using a 3D gradient-echo sequence (TR/TE, 3.9/1.9 ms; flip angle, 10°; FOV, 20 × 20 cm; matrix, 256 × 230; section thickness, 1.0 mm interpolated to 0.5 mm) 160 and 360 s, respectively, after the injection of contrast material.	dedicated 16-channel surface breast coi
Palshof	Annals of surgical oncolo gy	2021	1.5	GE	one axial T2W fast spin echo (FSE), one axial diffusionweighted imaging (DWI), and one T1W sequence before infusion of Multihance 0.2 ml/kg at an infusion rate 1.5 ml/ s. After admission of contrast, five T1W sequences (multiphase), one T1W sagittal, and one TW (with phase AP) including subtraction recordings were performed.	Liberty 9000 8 Breast coi
Winder	ANZ journal of surgery	2021	3.0	GE	pre-contrast axial T1, axial ideal and dynamic axial T1 (one phase pre and five phases post), three-dimensional fatsaturated post-contrast.	Dedicated breast coil

Supplementary Table 2: Search string used for obtaining relevant articles and abstracts on Pubmed and Embase.

Blocks<br>*combined with AND*	Pubmed	Embase
**Domain (1)**	((((breast cancer[Title/Abstract]) OR (breast neoplasm*[Title/Abstract])) OR (Breast carcinoma[Title/Abstract])) OR (Breast carcinomas[Title/Abstract])) OR (Breast Neoplasms[MeSH])	'breast cancer':ab,ti,kw OR 'breast neoplasm*':ab,ti,kw OR 'breast carcinoma':ab,ti,kw OR 'breast carcinomas':ab,ti,kw OR 'breast cancer'/exp OR 'breast carcinoma'/exp
**Domain (2)**	((((“Neoadjuvant Therapy"[Mesh]) OR (Neoadjuvant[Title/Abstract])) OR (Neo adjuvant[Title/Abstract])) OR (Preoperative[Title/Abstract])) OR (Pre operative[Title/Abstract])	'neoadjuvant therapy'/exp OR 'neoadjuvant chemotherapy'/exp OR 'neoadjuvant':ab,ti,kw OR 'neo adjuvant':ab,ti,kw OR 'preoperative':ab,ti,kw OR 'pre operative':ab,ti,kw
**Determinant**	((((Magnetic Resonance Imaging[Mesh]) OR (Magnetic resonance imaging[Title/Abstract])) OR (MRI[Title/Abstract])) OR (Radiologic* response[Title/Abstract])))	'nuclear magnetic resonance imaging'/exp OR ‘magnetic resonance imaging’:ab,ti,kw OR ‘MRI’:ab,ti,kw OR ‘radiologic* response’:ab,ti,kw
**Outcome**	(((((((((((((((((Pathological response*[Title/Abstract]) OR (Pathologic response*[Title/Abstract])) OR (Pathological complete response*[Title/Abstract])) OR (Pathologic complete response*[Title/Abstract])) OR (pCR[Title/Abstract])) OR (residual cancer burden[Title/Abstract])) OR (RCB[Title/Abstract])) OR (therapeutic effect[Title/Abstract])) OR (therapeutic effects[Title/Abstract])) OR (response to treatment[Title/Abstract])) OR (response to treatments[Title/Abstract])) OR (therapeutic response[Title/Abstract])) OR (therapeutic responses[Title/Abstract])) OR (treatment effect[Title/Abstract])) OR (neoplasm, residual[MeSH])) OR (Treatment Outcome[MeSH])) OR (residual tumor[Title/Abstract])) OR (residual tumour[Title/Abstract])) OR (treatment response[Title/Abstract])) OR (complete response[Title/Abstract])	‘pathological response*’:ab,ti,kw OR ‘pathologic response*’:ab,ti,kw OR ‘pathological complete response*’:ab,ti,kw OR ‘pathologic complete response*’:ab,ti,kw OR 'pathologic complete response'/exp OR ‘pCR’:ab,ti,kw OR ‘residual cancer burden’:ab,ti,kw OR ‘RCB’:ab,ti,kw OR ‘therapeutic effect’:ab,ti,kw OR 'therapy effect'/exp OR ‘therapeutic effects’:ab,ti,kw OR ‘response to treatment’:ab,ti,kw OR ‘response to treatments’:ab,ti,kw OR ‘therapeutic response’:ab,ti,kw OR ‘therapeutic responses’:ab,ti,kw OR 'treatment response'/exp OR ‘treatment effect’:ab,ti,kw OR 'minimal residual disease'/exp OR 'treatment outcome'/exp OR ‘residual tumor’:ab,ti,kw OR ‘treatment response’:ab,ti,kw OR ‘complete response’:ab,ti,kw OR 'complete response'/exp

Supplementary Figure 1: Risk of bias assessment of included studies based on QUADAS-2 criteria

(a) Summary plot total risk of bias per category (b) traffic light plot: risk of bias per category in each individual study

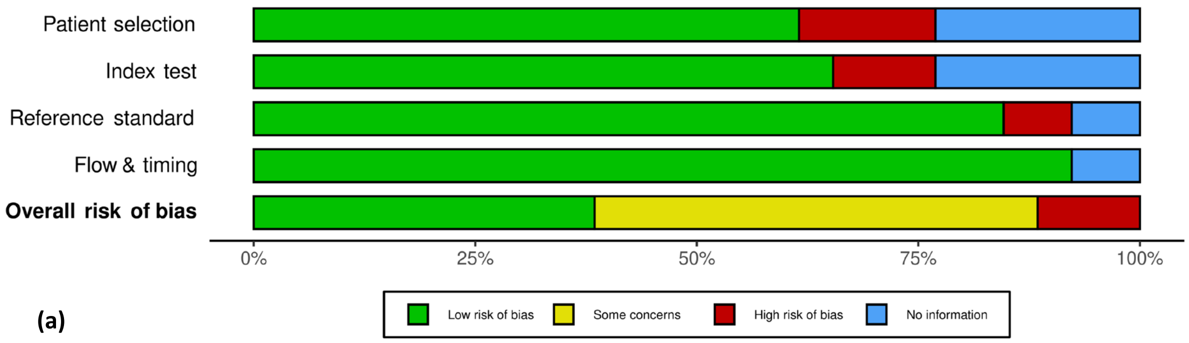

(a)

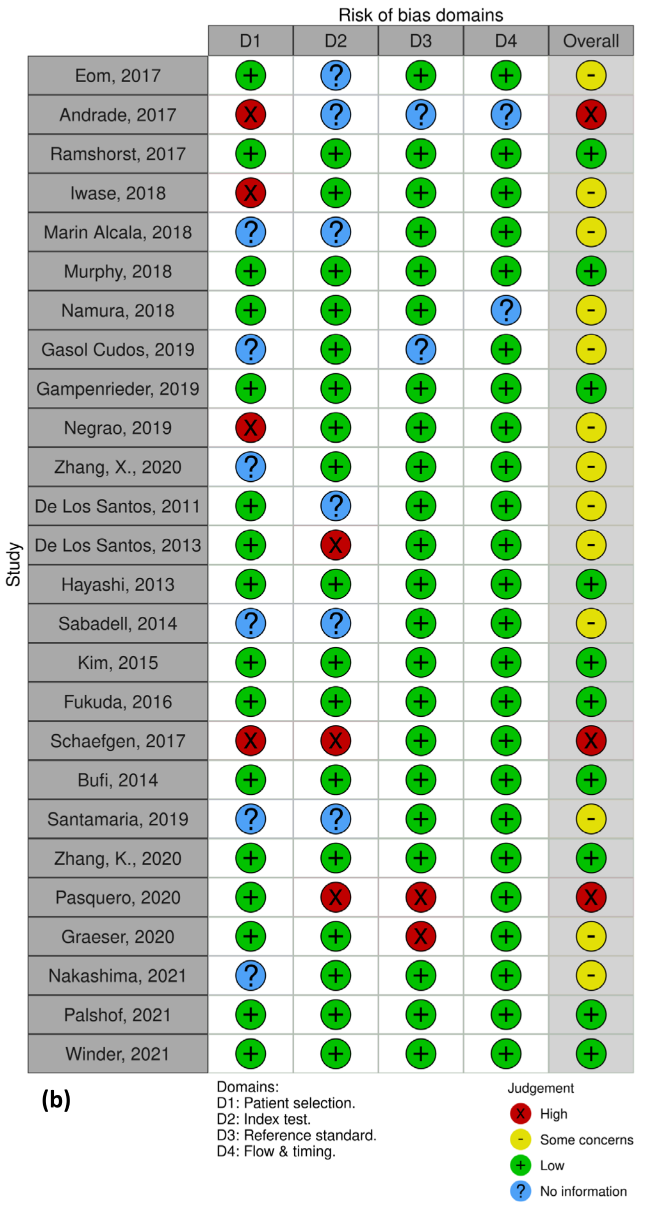

(b)

Supplementary Figure 2: Forest plots for pooled sensitivity and specificity with their 95% confidence intervals of MRI for pCR in each of the breast cancer IHC subtypes (a) HR-/HER2- subtype (b) HR-/HER2+ subtype (c) HR+/HER2- (d) HR+/HER2+ subtype

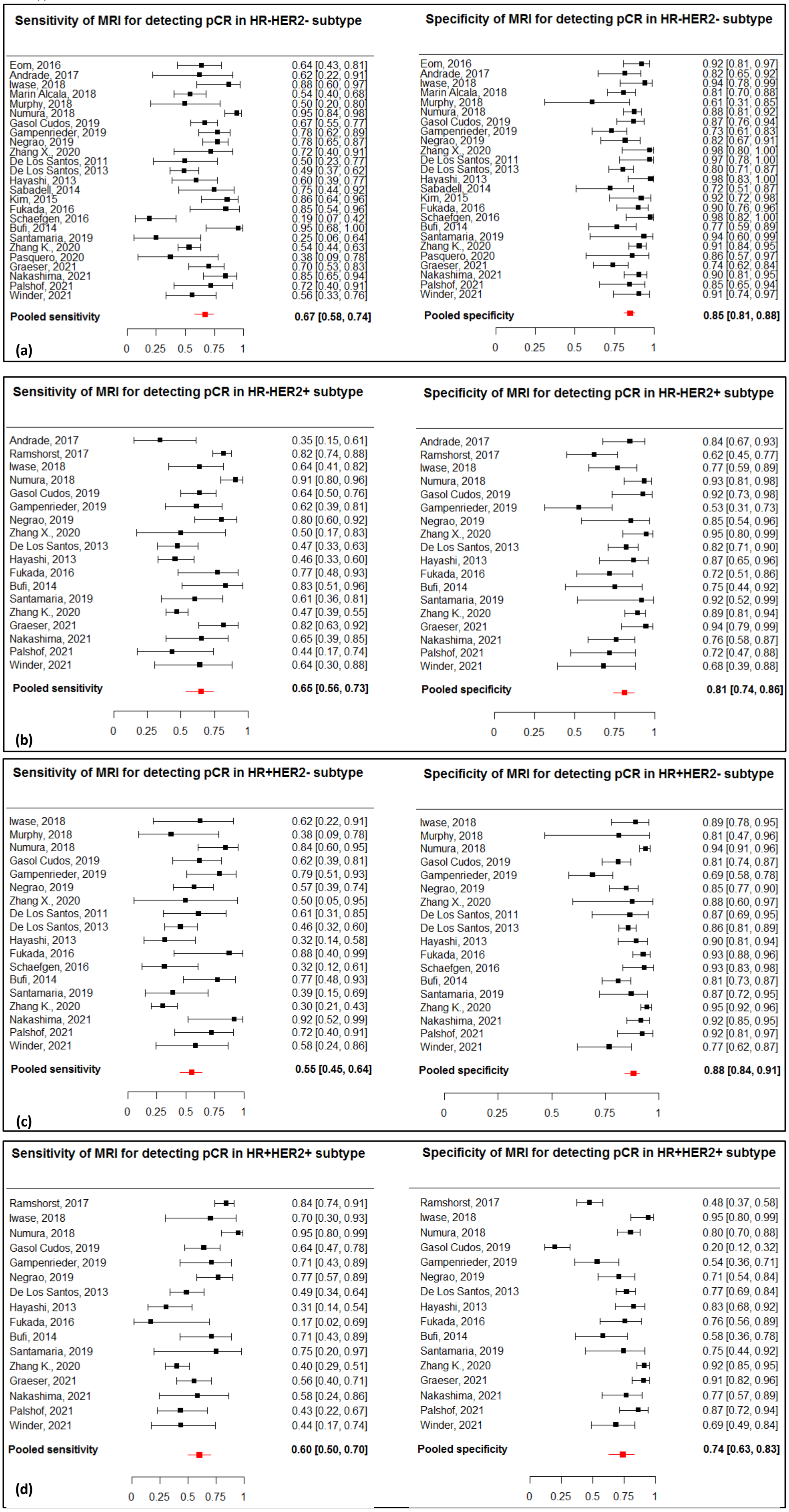

Supplementary Figure 3: Deeks funnel plots per IHC subtype (a) HR+/HER2- subtype (b) HR+/HER2+ subtype (c) HR-/HER2+ subtype (d) HR-/HER2+ subtype

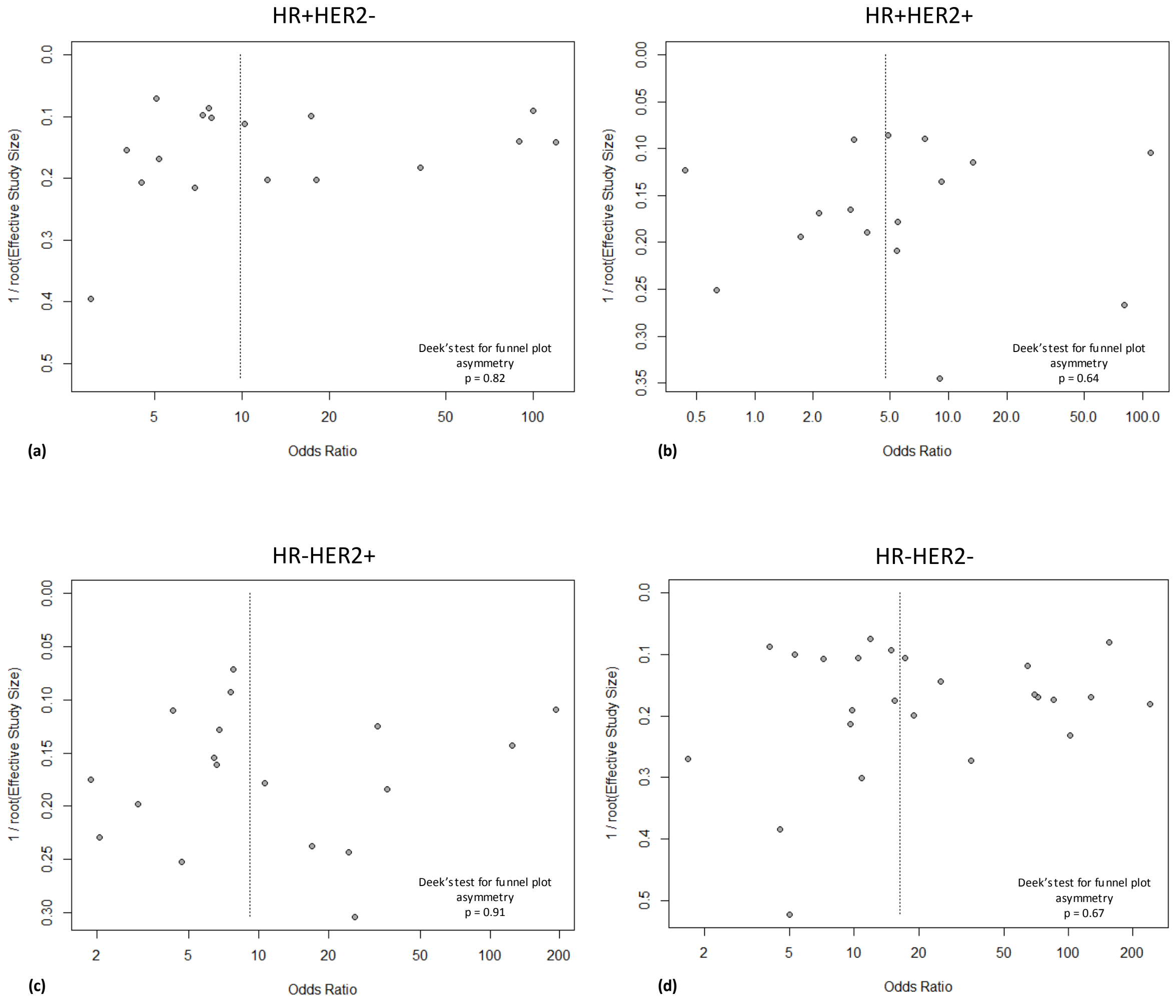

Supplement: Supplementary file 1 — Supplementary Material [file 41523_2022_475_MOESM1_ESM.pdf]
