# Supplementary material for: MRI to assess response after neoadjuvant chemotherapy in breast cancer subtypes: a systematic review and meta-analysis
Source: NPJ Breast Cancer. 2022 Sep 19;8:107. doi: 10.1038/s41523-022-00475-1 (PMC9485124; doi:10.1038/s41523-022-00475-1)
Supplement: Supplementary file 2 — Reporting Summary [file 41523_2022_475_MOESM2_ESM.pdf]

nature research

Corresponding author(s): L.M. Janssen

Last updated by author(s): 18-7-2022

Reporting Summary

Nature Research wishes to improve the reproducibility of the work that we publish. This form provides structure for consistency and transparency in reporting. For further information on Nature Research policies, see our Editorial Policies and the Editorial Policy Checklist.

Statistics

For all statistical analyses, confirm that the following items are present in the figure legend, table legend, main text, or Methods section.

n/a	Confirmed	
☐	☒	The exact sample size (n) for each experimental group/condition, given as a discrete number and unit of measurement
☒	☐	A statement on whether measurements were taken from distinct samples or whether the same sample was measured repeatedly
☐	☒	The statistical test(s) used AND whether they are one- or two-sided<br>*Only common tests should be described solely by name; describe more complex techniques in the Methods section.*
☐	☒	A description of all covariates tested
☐	☒	A description of any assumptions or corrections, such as tests of normality and adjustment for multiple comparisons
☐	☒	A full description of the statistical parameters including central tendency (e.g. means) or other basic estimates (e.g. regression coefficient) AND variation (e.g. standard deviation) or associated estimates of uncertainty (e.g. confidence intervals)
☐	☒	For null hypothesis testing, the test statistic (e.g. F, t, r) with confidence intervals, effect sizes, degrees of freedom and P value noted<br>*Give P values as exact values whenever suitable.*
☒	☐	For Bayesian analysis, information on the choice of priors and Markov chain Monte Carlo settings
☒	☐	For hierarchical and complex designs, identification of the appropriate level for tests and full reporting of outcomes
☐	☒	Estimates of effect sizes (e.g. Cohen's d, Pearson's r), indicating how they were calculated

Our web collection on statistics for biologists contains articles on many of the points above.

Software and code

Policy information about availability of computer code

Data collection	IBM SPSS Statistics 26.0.0.1 was used to store collected data from the individual studies
Data analysis	Statistical analysis were performed with R software, mainly using the functions 'reitsma' and 'forest' from package 'mada' and the function 'metabin' and 'funnel' from package 'meta'.

For manuscripts utilizing custom algorithms or software that are central to the research but not yet described in published literature, software must be made available to editors and reviewers. We strongly encourage code deposition in a community repository (e.g. GitHub). See the Nature Research guidelines for submitting code & software for further information.

Data

Policy information about availability of data

All manuscripts must include a data availability statement. This statement should provide the following information, where applicable:

- Accession codes, unique identifiers, or web links for publicly available datasets
- A list of figures that have associated raw data
- A description of any restrictions on data availability

The data collection forms, datasets containing extracted data and datasets used for analysis and R code are available from the corresponding author on reasonable request.

Field-specific reporting

Please select the one below that is the best fit for your research. If you are not sure, read the appropriate sections before making your selection.

☒ Life sciences ☐ Behavioural & social sciences ☐ Ecological, evolutionary & environmental sciences

For a reference copy of the document with all sections, see nature.com/documents/nr-reporting-summary-flat.pdf

Life sciences study design

All studies must disclose on these points even when the disclosure is negative.

Sample size	No sample size calculation was performed since this was dependant on the available studies for the meta-analysis.
Data exclusions	Studies were excluded from meta-analysis if they did not meet the inclusion criteria: 1) women with histopathologic proven early stage, invasive breast cancer treated with NAC; 2) patients who underwent MRI after NAC to assess response to treatment before surgery; 3) patients have undergone breast surgery after completion of NAC and data on pCR are documented; 4) original research article; 5) sufficient data to reconstruct two-by-two (2x2) table per IHC subtype. Studies investigating breast cancer in men were excluded because of the difference in presentation on MRI. If one study or cohort was reported by more than one publication, only the most informative publication was included. Articles that only reported on one IHC subtype were included.
Replication	Study selection and data extraction were performed by 2 independent researchers, data and code are available at request.
Randomization	not applicable
Blinding	the two independant reseachers were blinded for each others results when selecting studies and extracting data.

Reporting for specific materials, systems and methods

We require information from authors about some types of materials, experimental systems and methods used in many studies. Here, indicate whether each material, system or method listed is relevant to your study. If you are not sure if a list item applies to your research, read the appropriate section before selecting a response.

Materials & experimental systems

n/a	Involved in the study	
☒	☐	Antibodies
☒	☐	Eukaryotic cell lines
☒	☐	Palaeontology and archaeology
☒	☐	Animals and other organisms
☒	☐	Human research participants
☒	☐	Clinical data
☒	☐	Dual use research of concern

Methods

n/a	Involved in the study	
☒	☐	ChIP-seq
☒	☐	Flow cytometry
☒	☐	MRI-based neuroimaging
